# Identification of Loci Controlling Restriction of Parasite Growth in Experimental *Taenia crassiceps* Cysticercosis

**DOI:** 10.1371/journal.pntd.0001435

**Published:** 2011-12-20

**Authors:** Ruben Ramirez-Aquino, Irena Radovanovic, Anny Fortin, Edda Sciutto-Conde, Gladis Fragoso-González, Philippe Gros, Irma Aguilar-Delfin

**Affiliations:** 1 Departament of Immunology, Instituto de Investigaciones Biomédicas, Universidad Nacional Autónoma de México, Ciudad de México, México; 2 Department of Biochemistry, McGill University, Montreal, Canada; 3 Laboratory of Immunogenomics and Metabolic Diseases, Instituto Nacional de Medicina Genómica (INMEGEN), Ciudad de México, México; Imperial College, United Kingdom

## Abstract

Human neurocysticercosis (NC) caused by *Taenia solium* is a parasitic disease of the central nervous system that is endemic in many developing countries. In this study, a genetic approach using the murine intraperitoneal cysticercosis caused by the related cestode *Taenia crassiceps* was employed to identify host factors that regulate the establishment and proliferation of the parasite. A/J mice are permissive to *T. crassiceps* infection while C57BL/6J mice (B6) are comparatively restrictive, with a 10-fold difference in numbers of peritoneal cysticerci recovered 30 days after infection. The genetic basis of this inter-strain difference was explored using 34 AcB/BcA recombinant congenic strains derived from A/J and B6 progenitors, that were phenotyped for *T. crassiceps* replication. In agreement with their genetic background, most AcB strains (A/J-derived) were found to be permissive to infection while most BcA strains (B6-derived) were restrictive with the exception of a few discordant strains, together suggesting a possible simple genetic control. Initial haplotype association mapping using >1200 informative SNPs pointed to linkages on chromosomes 2 (proximal) and 6 as controlling parasite replication in the AcB/BcA panel. Additional linkage analysis by genome scan in informative [AcB55xDBA/2]F1 and F2 mice (derived from the discordant AcB55 strain), confirmed the effect of chromosome 2 on parasite replication, and further delineated a major locus (LOD = 4.76, p<0.01; peak marker *D2Mit295*, 29.7 Mb) that we designate *Tccr1* (*T. crassiceps cysticercosis restrictive* locus *1*). Resistance alleles at *Tccr1* are derived from AcB55 and are inherited in a dominant fashion. Scrutiny of the minimal genetic interval reveals overlap of *Tccr1* with other host resistance loci mapped to this region, most notably the defective *Hc/C5* allele which segregates both in the AcB/BcA set and in the AcB55xDBA/2 cross. These results strongly suggest that the complement component 5 (C5) plays a critical role in early protective inflammatory response to infection with *T. crassiceps*.

## Author Summary

Infection with the cestode *Taenia solium* causes cysticercosis in humans and pigs. Neurocysticercosis is a severe manifestation of *T. solium* infection that constitutes an important health concern in developing countries. Studies in humans living in areas of endemic disease and in pigs experimentally infected have suggested a large spectrum of permissiveness to *T. solium* multiplication, with the possible contribution of genetic factors. In the present report, we have used an experimental mouse model of intraperitoneal infection with *Taenia crassiceps* to study the potential role of genetic factors in regulating replication of this parasite. Our study focused on two inbred mouse strains A/J and C57BL/6J that are respectively permissive and non-permissive to intraperitoneal multiplication of *T. crassiceps*. We have used a set of AcB/BcA recombinant congenic strains of mice along with standard F2 crosses to decipher the complexity and nature of the genetic component of the A/J vs. C57BL/6J interstrain difference in permissiveness. Our results point to a major role of the complement component 5 (C5) in early response and protection against *T. crassiceps* infection.

## Introduction

*Taenia solium* seriously affects human health in many countries of Latin America, Asia and Africa [1]. The life cycle of *T. solium* includes a larval phase (cysticercus), which develops in both pigs and humans from ingested eggs contaminating the environment. When humans ingest improperly cooked pork meat infected with live cysticerci, the cysticerci develop to the stage of an adult intestinal tapeworm, which produces millions of eggs that are then shed to the environment in human faeces [2]. In rural communities where the disease is endemic, unsanitary conditions and presence of free-roaming pigs result in up to 9% of the human open population of these areas to be infected. Despite this high infection rate, only a small fraction of carriers become symptomatic and develop NC, suggesting intrinsic differences in host susceptibility to infection and pathogenesis of the disease [3]. Indeed, several reports have pointed at possible genetic effects in response to cysticercosis in human and pigs. In humans, multi-case families were identified in areas of highly endemic disease, favoring the idea of the participation of multiple genes in NC causality [3]. In a case-control study, resistance to NC was found associated to HLA [4]. Also, a three to five fold difference in parasite load was detected in a genetically heterogeneous pig cohort experimentally challenged with *T. solium* eggs [5].

*Taenia crassiceps* is a tapeworm of wild and domestic animals, which does not cause clinical disease in non-immunocompromised humans [6]. *T. crassiceps* has been used as an experimental model for cysticercosis due to its ability to proliferate by budding [7] and colonize the peritoneal cavity of the murine host [7], where its replication can be measured over time by enumeration of recovered metacestodes. Although the *T. crassiceps* ORF strain is unable to develop into adult tapeworms [8], its property to rapidly multiply in the peritoneal cavity of infected mice has been extensively used to explore the relevance of biological factors in host-parasite interactions [9], and to identify protective antigens of interest for vaccine development [9], [10]. The mechanisms involved in the protective immunity against *T. crassiceps* cysticercosis have been extensively studied, but are not fully understood. Studies in inbred mouse strains (growth permissive H2d-bearing BALB/c; growth restrictive H2b-bearing C57BL/6J) initially pointed at the importance of the major histocompatibility locus (MHC) and MHC-linked genes in regulating intraperitoneal growth of the parasite [11]. This was confirmed by additional studies of H2 congenic BALB/c substrains, where BALB/cJ mice express the Qa2 protein and are significantly more resistant than the BALB/cAnN mice [12], [13]. This differential susceptibility may be explained in part by activation of antigen presenting cells, and production of pro-inflammatory cytokine and modulatory chemokines both early and late during *T. crassiceps* infection [14]. Furthermore, phenotyping of different inbred strains has suggested that an additional, non-MHC linked genetic component may contribute to regulation of *T. crassiceps* replication [15]. Finally, clear differences between the parasite load of male and female have been noted in inbred mouse strains [16]. Females show higher numbers of cysticerci compared to males due to a significant effect of sex hormones on response to infection [17], [18].

With the aim of further characterizing the host genetic factors that affect host response to *T. crassiceps* cysticercosis, the differential susceptibility of A/J (permissive) and C57BL/6J (restrictive) mouse strains was studied. For this, a set of 34 reciprocal AcB/BcA recombinant congenic strains (RCS) derived by systematic inbreeding from a double backcross (N3) between A/J and C57BL/6J parents [19] was phenotyped for response to *T. crassiceps* infection. In the breeding scheme used to derive the AcB/BcA strains set, each of the strains harbors 12.5% of its genome from either A/J or B6, fixed as a set of discrete congenic segments onto 87.5% of the reciprocal parental background. The vast range of permissiveness to *T. crassiceps* growth in 34 RCS, as measured by the parasite load 30 days post-infection, along with haplotype association mapping suggested that response to *T. crassicep*s cestode is under complex genetic control, with identifiable contributions of chromosomes 2 and 6. Subsequent genetic linkage analysis in informative crosses validated the chromosome 2 locus, and established the regional position of the regulating locus.

## Materials and Methods

### Mice

The AcB/BcA set of recombinant congenic strains (RCS) were derived from a double backcross (N3) between A/J and C57BL/6J parents at McGill University and were provided by Emerillon Therapeutics. The breeding, genetic characteristics and genotype of RCS have been described earlier [19]. Inbred strains A/J, B6, and DBA/2 were obtained as pathogen-free mice at 7–8 weeks of age from the Jackson Laboratory (Bar Harbor, ME) and maintained as breeding colonies at UNAM. [AcB55xDBA/2] F2 progeny were bred by systematic brother-sister mating of [AcB55xDBA/2] F1 mice.

### Ethics statement

The study protocol (register number 021) was approved by the ethics committee of the Instituto de Investigaciones Biomédicas, Universidad Nacional Autónoma de México (UNAM). All housing and experimental procedures were performed according to the principles set forth in the Guide for the Care and Use of Laboratory Animals, Institute of Laboratory Animal Resources, National Council, Washington, D.C. 1996.

### Infection with *Taenia crassiceps*

The fast growing ORF strain of *T. crassiceps*, originally isolated by R. S. Freeman [7], was maintained by serial intraperitoneal (i. p.) passage in female BALB/cAnN mice, as previously described [13]. All experimental mice were inoculated intraperitoneally with 10 small (<2 mm) non-budding *T. crassiceps* larvae, re-suspended in sterile isotonic saline. Thirty days following infection, parasites were harvested from the peritoneal cavity and counted using a stereoscopic microscope [20] to determine the parasite burden. Organs inside the abdominal cavity were removed and carefully inspected for any remaining *T. crassiceps* larvae.

### Genotyping

Genomic DNA was isolated from tail clips of individual F2 mice collected at the time of sacrifice, as previously described [19]. A total of 185 female [AcB55xDBA/2]F2 mice were genotyped at the Centre for Applied Genomics (The Hospital for Sick Children, Toronto, Canada) using the Illumina Mouse Low Density Linkage panel containing 377 SNPs distributed across the genome, out of which 161 were polymorphic between AcB55 and DBA/2 strains. Additional microsatellite markers were obtained from the Mouse Genome Informatics Database (www.informatics.jax.org) and used for gap filling and fine mapping by a standard PCR-based method employing (α-^32^P) dATP labeling and separation on denaturing 6% polyacrylamide gels.

C5 status in the F2 mice was confirmed by RFLP analysis, as previously described [21]. Briefly, C5 fragment was amplified by PCR and digested with *Bsg* I, which recognizes a novel restriction site introduced by the 2-bp deletion in exon 6 of the *Hc* gene [21], [22]. The fragments were resolved on 2% agarose gel; the expected size for wild-type C5 was 446 bp, while the sizes for the samples containing the deletion were 318 and 126 bp.

### Linkage and association mapping

QTL mapping was performed using Haley-Knott multiple regression analysis [23] and the two-dimensional scan was performed using the two-QTL model. Empirical genome-wide significance was calculated by permutation testing (1000 tests). All linkage analysis was performed using R/qtl [24]. The detailed algorithm underlying the efficient mixed-model for association mapping has been previously published [25]. The EMMA algorithm is based on the mixed-model association where a kinship matrix accounting for genetic relatedness between inbred mouse strains is estimated and then fitted to the vector of the phenotype, thereby reducing false positive rates. EMMA is publically available as an R package implementation.

### Statistical analysis

An unpaired, two-tailed Student's t-test was used to establish significance of differences in mean parasite burden between mouse *Tccr1* and *C5* genotypes. These data were analyzed using GraphPad Prism 4.0 statistical software. P-values<0.05 were considered significant.

## Results

### Response to *T. crassiceps* infection in recombinant congenic strains

**Figure 1:**
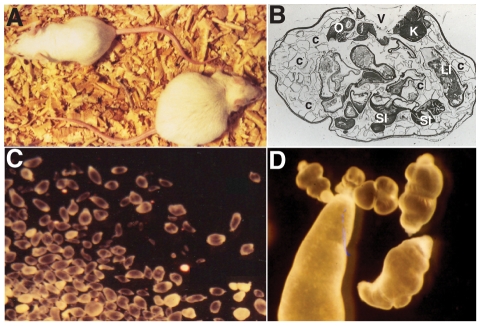
*Taenia crassiceps* intraperitoneal murine cysticercosis. Aspect of the distended abdomen of a cysticercotic permissive mouse 30 days after intraperitoneal *T. crassiceps* infection compared to a non-infected littermate (**A**). Hematoxilin and eosin staining was performed on a cross-sectional slice of the abdomen of a cysticercotic mouse (**B**). Abdominal organs can be distinguished (O = ovary, K = kidney, SI = small intestine, LI = large intestine, V = vertebral column space), whereas cysticerci (denoted by c) appear as thin dark septa limiting the empty spaces. (**C**) Aspect of cysticerci recovered from the peritoneal cavity of an infected permissive mouse 30 days post-infection, with and without attached buds (**D**). Photographs kindly provided by Dr. Carlos Larralde.

**Figure 2:**
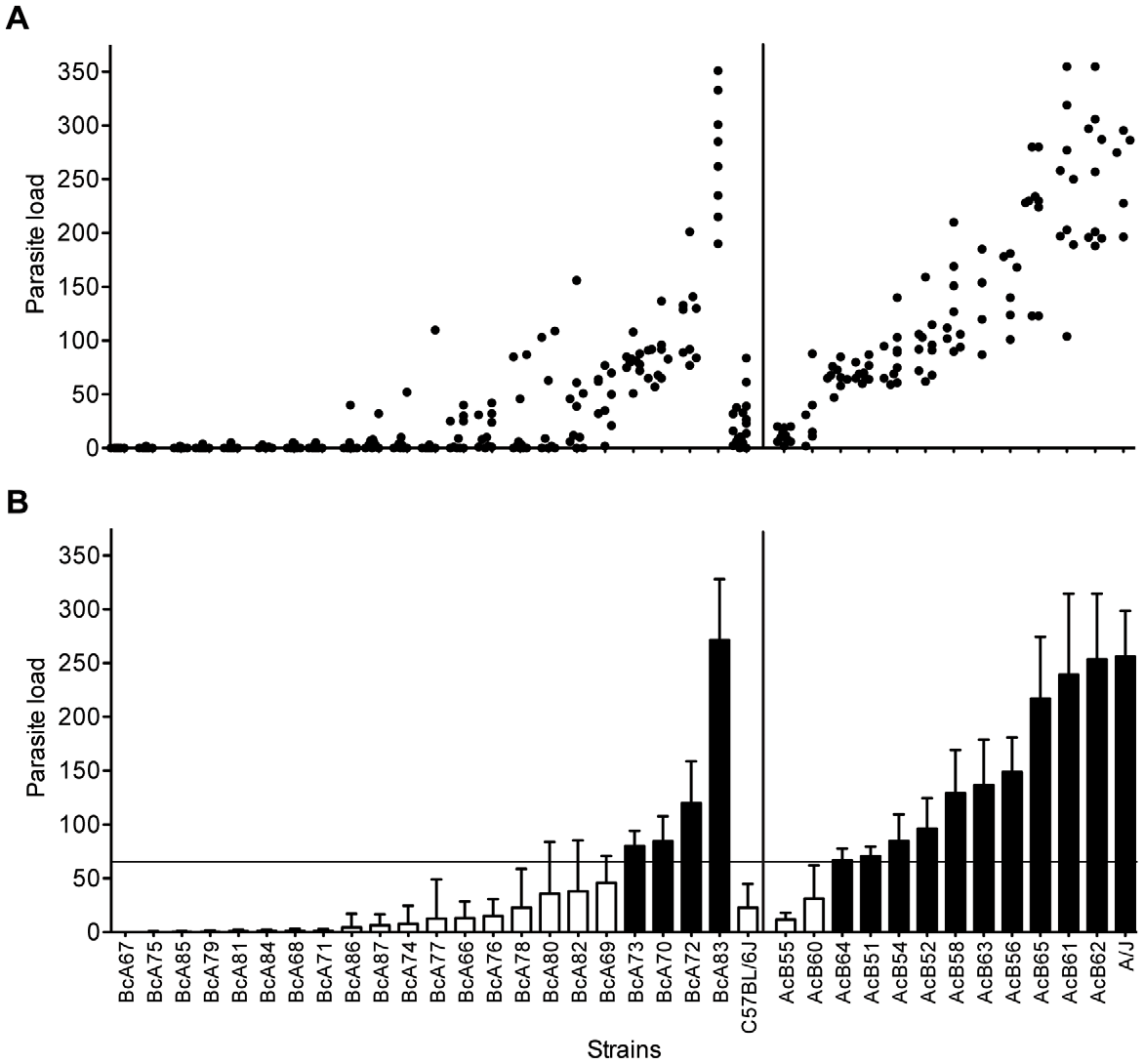
Response of AcB/BcA strains to *T. crassiceps* infection. Parasite load recovered from female A/J, C57BL/6J (B6) and 34 recombinant congenic AcB/BcA mice 30 days after infection. 4–10 mice per strain were intraperitoneally infected with 10 non-budding *T. crassiceps* larvae. The average number of parasites is shown (**B**) along with raw data (**A**) where each dot represents a single mouse. Bars represent strain mean ± SD. Horizontal line corresponds to two standard deviations from the mean parasite load of resistant B6 strain, which was used to stratify strains into susceptible (black bars), or resistant (white bars) categories.

A/J and C57BL/6J (B6) mice show differential permissiveness to cysticercosis [16], following the intra-peritoneal inoculation of 10 small (<2 mm diameter) non-budding *T. crassiceps* larvae (Figure 1). In A/J mice, there is rapid parasite reproduction in the peritoneal cavity, which is detectable by visual and histological examination of the mice (Figure 1A, 1B), and by quantification of parasite load (Figure 1C, magnification in 1D). Enumeration of the parasites recovered 30 days following infection (Figure 2) indicates a 10-fold difference in parasite replication between A/J (200–250) and C57BL/6J (15–30). To study the genetic control of differential replication of *T. crassiceps* in restrictive B6 and permissive A/J strains, we phenotyped a set of 34 AcB/BcA recombinant congenic strains [19]. The breeding scheme used to generate the reciprocal AcB/BcA strains set results in individual strains harboring 12.5% of its genome donated from either A/J (in BcA strains) or B6 (in AcB strains), fixed as a set of discrete congenic segments onto 87.5% of the reciprocal parental background [19]. The AcB/BcA strain set has previously been used to characterize different genetic traits that control phenotypic differences between B6 versus A/J, including mapping of major monogenic trait [19], and dissection of complex genetic traits into several simple genetic effects [26], [27]. Between 5–10 animals from each strain were infected with *T. crassiceps* and the total parasite burden was determined 30 days later (Figure 2A and 2B). We segregated AcB/BcA strains according to the predominant genetic background (left and right panels in Figure 2), and further grouped them into permissive or restrictive categories based on the overall mean parasitic load, whereby strains harboring an average of >66 parasites were deemed permissive while those showing <66 were termed restrictive (determined as two standard deviations from the parasite load of restrictive B6 parental group). According to this arbitrary segregation, the majority of BcA strains were parasite growth restrictive, with the notable exception of strains BcA73, 70, 72 and 83 that showed parasite loads similar to those detected in the A/J controls (Figure 2B). Conversely, AcB strains were found to be generally permissive for parasite growth, with the notable exception of strains AcB55 and AcB60 that displayed an average of 12 and 31 cysticerci, respectively. The presence of such discordant strains in both sets of RCS suggests the possibility that the restrictiveness/permissiveness trait is under simple genetic control, and that transfer of a single congenic fragments onto the opposite strain background strongly influences the phenotype of the recipient strain. Such a situation would be similar to the segregation of the *Ccs3* (colorectal cancer) [28], *Ity* (susceptibility to *Salmonella*) [29] and *Lgn1* loci (susceptibility to *Legionella*) [19] we previously reported in these strains.

### Chromosomes 2 and 6 are associated with response to *T. crassiceps* infection in AcB/BcA strains

**Figure 3:**
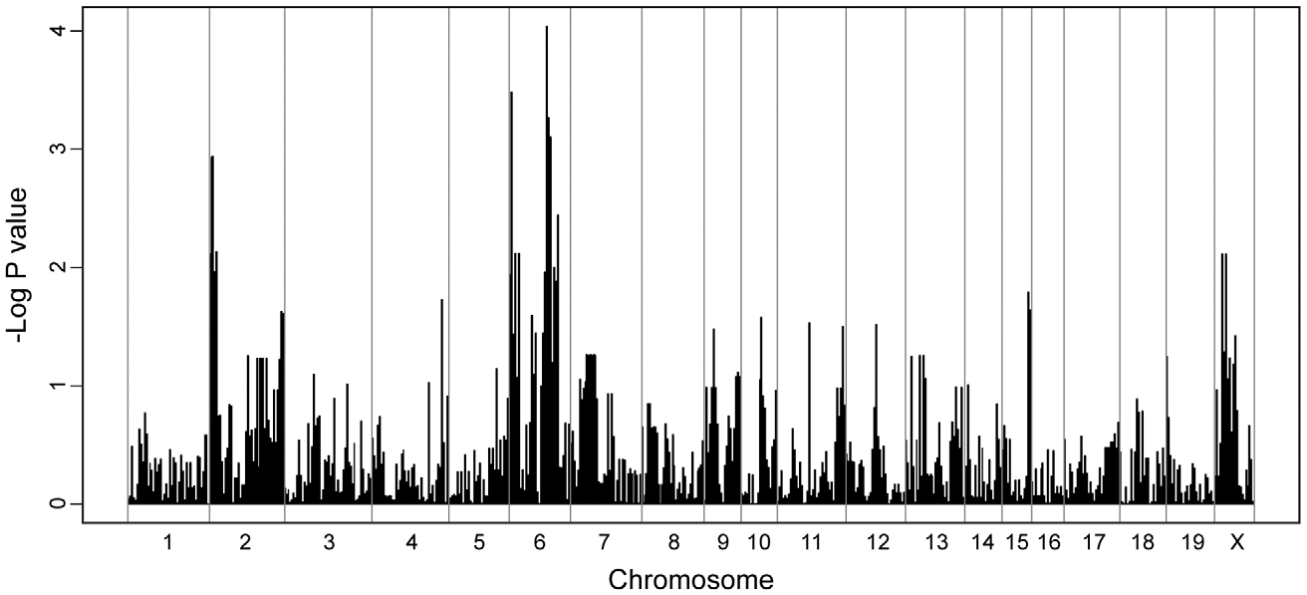
Genome-wide association mapping in 34 AcB/BcA strains using EMMA. Using the R package implementation of EMMA, genome-wide association mapping was conducted across 34 RCS as well as the original parental A/J and B6 inbred strains. The genotyping data consisted of ∼1200 SNPs and informative dinucleotide repeats and was correlated with the parasite load, yielding −log_10_ transformed P values indicating genome-wide association significance for each SNP. Each line represents a single SNP or marker.

To explore the nature and complexity of the genetic control of parasite replication in the AcB/BcA strains set, we performed haplotype association mapping using parasite load as the primary phenotype and 1200 informative polymorphic genetic markers. We applied a statistical model EMMA [25] that corrects for genetic relatedness and population structure of the RCS by computing a kinship matrix in a manner analogous to an inbred mouse strain analysis, as we previously described [30]. Using this approach, we detected suggestive association of chromosome 2 (proximal region) and chromosome 6 alleles with *T. crassiceps* permissiveness (Figure 3). In the case of chromosome 6, both proximal (weaker) and distal (stronger) portions of the chromosome showed association. Also, for both chromosomes 2 and 6, A/J alleles are associated with permissiveness while B6 alleles are associated with restrictiveness, as expected. Additional strength for these associations is provided by some of the phenodeviant strains; for example, for the proximal part of chromosome 2 (∼35 Mb), susceptible BcA70, 72, and 73 harbor A/J haplotypes, while resistant AcB55 and AcB60 harbor B6-derived haplotypes (Figure S1). Likewise, for distal chromosome 6, BcA72, 73 and 83 have A/J alleles, while AcB55 and 60 have B6 alleles (Figure S1). Nevertheless, the imperfect correlation for both chromosomes requires validation in secondary crosses. It also suggests presence of additional genetic effects controlling *T. crassiceps* permissiveness in the strain set.

### Linkage analysis in [AcB55xDBA/2]F2 cross validates association of chromosome 2 locus with response to *T. crassiceps*

**Figure 4:**
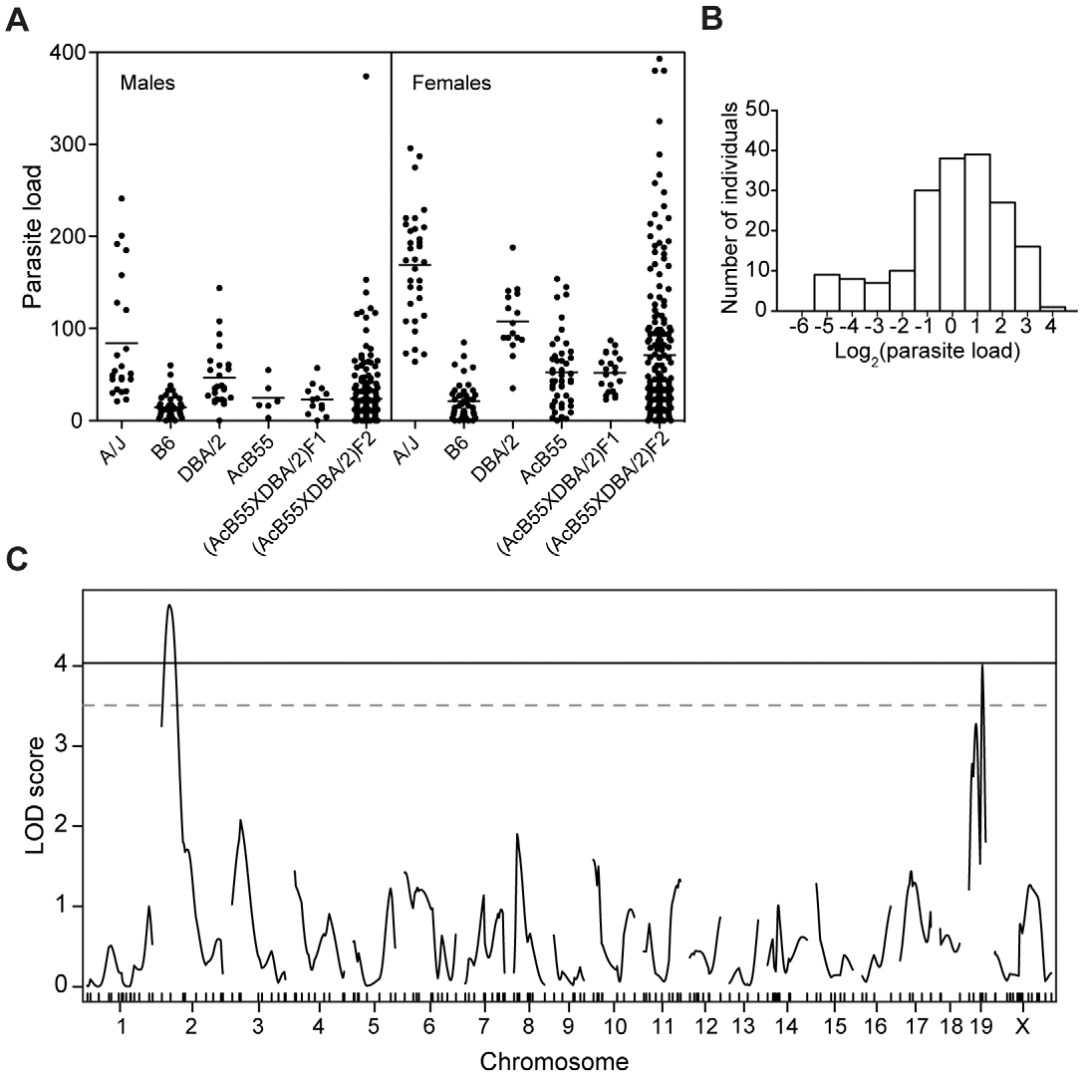
Linkage analysis in the informative [AcB55xDBA/2]F2 population. [AcB55xDBA/2]F2 mice (n = 379) were infected intraperitoneally with 10 non-budding *T. crassiceps* larvae and parasite number was determined 30 days post-infection. The results from three separate infections are plotted along with A/J, B6, DBA/2, and [AcB55xDBA/2]F1 controls (**A**). Each dot represents a single mouse. Distribution of parasite load is shown in the female [AcB55xDBA/2]F2 population (**B**), after regression of log_2_(parasite load) to an experiment-specific mean (set at 0). Mice were genotyped at 171 SNPs and dinucleotide repeats across the genome and interval mapping was carried out using the R/qtl software package. Whole genome LOD score trace is shown for genetic effects controlling parasite burden in female [AcB55xDBA/2]F2 mice (n = 185) (**C**), identifying linkage to chromosome 2 (LOD = 4.75) and chromosome 19 (LOD = 4.0), designated *Tccr1* and *Tccr2*, respectively. Marker positions are indicated on the x-axis and genome-wide thresholds at P = 0.01 and 0.05 were identified by permutation testing (1000 tests).

The genetic control of host response to *T. crassiceps* was further investigated in strain AcB55. This strain consistently showed the lowest parasite burden in the AcB set (Figure 2, mean parasite load = 11.7), despite ∼87.5% of its background being inherited from the highly susceptible A/J parent (mean parasite load = 256). Therefore, we hypothesized that AcB55 is likely to carry B6-derived chromosomal segments responsible for restrictiveness in both AcB55 and B6. To map such segments, AcB55 was crossed to the permissive strain DBA/2 (Figure 4A, mean parasite load = 108) to produce an [AcB55xDBA/2] F2 population in which individual animals would be informative for the entire genome in linkage analyses. [AcB55xDBA/2] F1 hybrids and 379 [AcB55xDBA/2] F2 animals were infected intraperitoneally with 10 non-budding *T. crassiceps* larvae in three separate infections, and parasite burden was measured 30 days later (Figure 4A). Due to the previously reported gender-associated differential permissiveness to *T. crassiceps*, where higher concentrations of estrogen and estradiol are concomitant with increased parasite burdens [17], [18] typically occurring in female mice, we segregated males and females in the analysis (Figure 4A). Both male and female [AcB55xDBA/2] F1 hybrids were fully resistant with parasite load similar to the AcB55 controls (Figure 4A), suggesting that resistance to *T. crassiceps* is inherited in a dominant fashion. The parasite load of [AcB55xDBA/2] F2 mice followed a continuous distribution between highly permissive and highly restrictive animals, with a clustering of F2 animals in the resistant range. This suggests both a complex genetic control of permissiveness to infection, with a dominant pattern of inheritance of restrictiveness more apparent in the female population (Figure 4A). Interestingly, we observed an identical pattern of inheritance of restrictiveness (dominant) in a distinct F2 population, where the AcB55 strain was crossed to the permissive A/J founder (Figure S2). However, due to limited genetic diversity in the [AcB55xA/J] F2 progeny (∼12.5% due to B6 genomic segments), we conducted genetic linkage analysis by whole genome scanning in the [AcB55xDBA/2] F2 population. Because the frequency distribution of parasite load in F2 females was skewed, we applied a log2 correction to normalize the distribution, followed by regression to an experiment-specific mean (set at 0) (Figure 4B).

**Figure 5:**
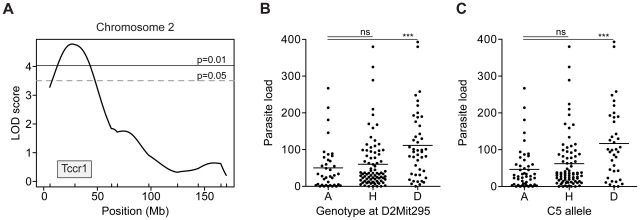
Effect of the *Tccr1* locus on parasite burden in [AcB55xDBA/2]F2 mice. Detailed LOD score traces are shown for chromosome 2 (*Tccr1*) locus for [AcB55xDBA/2]F2 females (**A**). The shaded area designates the Bayesian 95% confidence interval and the genome-wide thresholds are indicated at P = 0.01 and P = 0.05. The effect of the AcB55 (A), DBA/2 (D) or heterozygous (H) allele on parasite load is depicted for *Tccr1* (**B**) at the D2Mit295 peak marker and for the underlying *C5* gene (**C**).

A total of 185 [AcB55xDBA/2] F2 female mice were genotyped with the Illumina Mouse Low Density Linkage Panel consisting of 161 informative polymorphic markers with 10 additional microsatellite markers to complete genome coverage. Whole-genome multiple regression linkage analysis in R/qtl (Figure 4C) identified a highly significant locus associated with parasite burden on chromosome 2 (LOD = 4.76, P<0.01) and an additional suggestive linkage on chromosome 19 (LOD = 4.03, P<0.05) (Figure 4C and S3). These loci contributing restrictiveness to *T. crassiceps* in the AcB55 strain were given a temporary appellation *Tccr1* (*Taenia crassiceps cysticercosis restrictiveness 1*) for chromosome 2 QTL and *Tccr2*, for chromosome 19 QTL. The Bayesian 95% credible intervals were determined to be 13.1–44.1 Mb for *Tccr1* (Figure 5A) and the entire chromosome 19 for *Tccr2* (Figure S3), whereas the peak LOD scores were identified at 29.7 Mb (peak marker: D2Mit295) and 46.7 Mb (peak SNP: rs13483650), respectively.

To examine the effect of the most significant *Tccr1* locus on parasite load, F2 mice were segregated according to genotype at the most significant chromosome 2 marker *D2Mit295* (Figure 5B). Mice carrying the DBA/2 alleles at *Tccr1* have significantly higher number of cysticerci (P = 0.0009; Student's t-test) than those harboring one or two AcB55 alleles. This indicates that restrictiveness alleles at *Tccr1* are inherited in a strictly dominant fashion. We also examined the LOD score trace for chromosome 19 and the inheritance of parental alleles underlying *Tccr2* at the peak marker (rs13483650) (Figure S3). We observed two prominent peaks, suggesting that *Tccr2* may be due to multiple genetic effects contributing to lower parasite loads in the AcB55 strain. However, once we segregated the female F2 mice according to their genotype (Figure S3B), we observed that the *Tccr2* QTL is driven by heterozygosity, with homozygosity for neither the AcB55- nor the DBA/2-derived alleles having a significant effect on the parasite burden. To determine whether *Tccr1* and *Tccr2* acted in an additive or epistatic manner, a two-dimensional Haley-Knott multiple regression analysis was carried out, followed by simulation of an overall QTL model using R/qtl. This analysis revealed that, although *Tccr1* and *Tccr2* are non-interacting loci, their individual and joint contribution to the full QTL model raises the LOD score to 11.26 and explains 25% of observed phenotypic variance.

Interestingly, linkage analysis in the AcB55xDBA/2 cross was successful in validating the chromosome 2 association initially detected by EMMA analysis in 34 RCS (Figure 3), but not the more significant chromosome 6 association. In fact, detailed examination of the region underlying chromosome 6 indicated that both AcB55 and DBA/2 strains harbor similar haplotype blocks at the proximal and distal regions of chromosome 6 (data not shown) and would therefore not segregate in the analyzed F2 cross. Together, haplotype association mapping in 34 RCS along with linkage analysis in an informative F2 cross strongly suggest that *T. crassiceps* replication in the murine host is controlled by multiple genetic factors, amongst which *Tccr1* strongly contributes to the noted resistance of AcB55 mice.

### Hemolytic complement (*Hc/C5*) underlies *Tccr1*

Maximum linkage for *Tccr1* locus is coupled to the *D2Mit295* marker, which lies at 29.7 Mb and was previously associated to the gene coding for hemolytic complement (*Hc/C5*) [21] located approximately 5 Mb further downstream (34.8–34.9 Mb). Since *C5* was mapped in an F2 cross derived from A/J and B6 strains and its deficiency correlated to high susceptibility to the fungal pathogen *Candida albicans* in the majority of RCS [21], we examined the involvement of *C5* in the context of *T. crassiceps* infection. We genotyped the parental AcB55 and DBA/2 strains for the deficiency-causing 2-bp deletion in the *C5* gene and confirmed that DBA/2 is C5-deficient [22], while AcB55 is wild type for C5 and does not harbor the deletion [21]. C5 status was also determined in 185 female [AcB55xDBA/2] F2 mice and the parasite replication permissiveness was associated with *C5*-deficiency (Figure 5C) in a recessive manner, where mice harboring at least one functional copy of the gene are fully resistant. In addition, classifying the set of 34 RCS according to permissiveness to infection and C5 status further corroborates the association of wild-type C5 alleles with increased protection against *T. crassiceps* (Table 1).

**Table 1:** Phenotypic response of RCS to *T. crassiceps* infection, with respect to the C5 status.

RCS	Hc allele^a^	Parasite load(mean ± s. d.)	Phenotype^b^
BcA83	0	271.5±56.6	P
BcA73	0	80.1±14.2	P
BcA72	0	119.6±39.1	P
BcA70	0	84.6±23.2	P
AcB65	0	216.9±57.5	P
AcB64	0	66.9±10.8	P
AcB63*	1	136.5±42.4	P
AcB62	0	253.6±61.1	P
AcB61	0	239.1±75.5	P
AcB58	0	129.0±40.2	P
AcB56	0	148.7±32.4	P
AcB54	0	84.7±24.7	P
AcB52	0	96.4±28.0	P
AcB51	0	70.8±8.8	P
A/J	0	256.1±42.5	P
BcA87	1	6.6±10.0	R
BcA86	1	4.5±12.6	R
BcA85	1	0.3±0.7	R
BcA84	1	0.8±1.3	R
BcA82	1	38.1±47.2	R
BcA81	1	0.5±1.6	R
BcA80	1	35.8±48.3	R
BcA79	1	0.4±1.3	R
BcA78	1	22.9±36.1	R
BcA77	1	12.6±36.6	R
BcA76*	0	15.1±15.7	R
BcA75	1	0.2±0.6	R
BcA74	1	7.8±16.9	R
BcA71	1	1.0±1.8	R
BcA69	1	45.9±25.0	R
BcA68	1	1.0±2.0	R
BcA67	1	0.0±0.0	R
BcA66	1	13.0±15.4	R
B6	1	22.8±21.9	R
AcB60*	0	31.2±31.1	R
AcB55	1	11.7±6.4	R

34 RCS along with A/J and B6 controls are classified as permissive (P) or restrictive (R) to *T. crassiceps* infection. Discordant strains are denoted by *.

a 0-C5 deficient; 1-C5 sufficient.

b R-restrictive; P-permissive.

*Discordant strains.

Taken together our results suggest a critical role for the complement component 5 in restricting proliferation of *T. crassiceps* in mice.

## Discussion

The panel of 34 reciprocal AcB/BcA RCS has been used to map major monogenic traits [19], [28] or to facilitate identification of multiple loci involved in complex trait diseases [26], [27]. This approach is based on the premise that unique small congenic fragments derived from the donor strain are fixed and delineated for each strain, which may allow for detection of causative haplotype by the sole study of the strain distribution pattern in relation to the phenotype of interest [19]. Here, we have phenotyped the set of 34 RCS to study the genetic control of susceptibility to *T. crassiceps* cysticercosis (Figure 2), where the majority of AcB strains were found to be permissive for parasite replication and conversely, most of the BcA strains were deemed restrictive, similarly to progenitor strains A/J and B6, respectively. The presence of phenodeviant strains allowed us to conduct haplotype association mapping and identify significant genomic regions associated with response to infection on chromosomes 2 and 6 (Figure 3). Subsequent genome scan in informative F2 mice generated between resistant AcB55 and susceptible DBA/2 progenitors (fixed for chromosome 6 locus) identified a highly significant linkage on chromosome 2 (*Tccr1*, LOD score = 4.76, P value<0.01) as conferring resistance to AcB55 in a dominant fashion along with an additional heterozygous-driven effect observed on chromosome 19 (*Tccr2*, LOD score = 4.03, P value = 0.05) (Figure 4 and Figure 5).

Interestingly, the *Tccr1* locus was analogous to a previously identified susceptibility locus for the fungal pathogen *Candida albicans* attributable to the complement component 5 gene (*Hc/C5*) [21]. The complement pathway represents the initial line of defense of the innate immune system and elicits an inflammatory response to the site of infection [31], [32]. Activation of the complement cascade is triggered by microbial products via several pathways, which ultimately results in the activation of C3 convertase, cleavage of C5, release of chemotactic factors (C3a and C5a), and generation of the membrane attack complex (MAC) [31]. Similarly to *T. crassiceps* infection, inbred mouse strains display various degrees of susceptibility to *C. albicans*, where A/J is highly susceptible and B6 resistant [21]. This differential susceptibility was attributed to the major gene *C5*, where a single allele of the wild-type C5 confers complete resistance to infection [21]. As previously described [22], C5-deficiency is caused by a 2-bp deletion in the exon 6, leading to a premature stop codon and a product that is not secreted in the serum [33]. We have confirmed that AcB55 carries the B6 allele at C5 and is therefore C5-suficient and resistant to *T. crassiceps*, whereas DBA/2 is C5-deficient and susceptible. C5 status was also determined in 185 [AcB55xDBA/2]F2 progeny (Figure 5C), where resistance segregated with one or two copies of the wild-type C5 indicating that C5 exerts an early dominant protective effect upon *T. crassiceps* infection.

In the mouse model of *T. crassiceps* infection, although the extent of parasite replication depends on the orchestration of immune and hormonal responses, the parasite restriction is initiated by the early immunological response, which was shown to destroy the peritoneal larvae [34] and involve the complement system in innate resistance to *T. taeniaeformis* [35]. Earlier studies demonstrated that C3 along with IgG is deposited on larval *T. taeniaeformis* [35], [36] as early as 2 days post infection, but that it is not directly involved in lytic activity through formation of the MAC. Rather, the complement system indirectly triggers host cell recognition and/or activation [35], leading to parasite elimination. Inhibition of complement by cobra venom factor administration to the resistant BALB/cByJ mice prior to *T. taeniaformis* infection results in decreased parasite mortality, indicating that complement component deposition and activation is necessary in host defense against taenid infection [35]. More precisely, the role of complement component 5 (C5) was established in the mouse model of hyatid disease, caused by the *Echinococcus granulosus* cestode. C5-deficiency in B10.D2 o/SnJ mice infected with *E. granulosus* was associated with poor eosinophil infiltration and increased growth of established cysts [37], indicating that C5-mediated mechanisms are detrimental for parasite growth.

The immunopathology of *T. crassiceps* cysticercosis in mice is that of an initial non-permissive Th1 type, which shifts to a parasite permissive Th2 type during chronic stage of infection and is accompanied by an increase in IL-4, IL-6 and IL-10 cytokines [38]–[40]. This transition was also reported in individuals with *T. solium* neurocysticercosis [41] and more precisely, in the brain granulomas surrounding *T. solium* parasite [42]. Although A/J mice succumb to *C. albicans* infection early after infection (48 h), they mount a similar Th2-like cytokine storm consisting of high levels of IL-6, IL-10 and TNFα [43], [44], suggesting a common role for C5 in both pathologies. In addition, animals deficient for the Th1 hallmark cytokine IFNγ or upon its neutralization exhibit increased susceptibility to *C. albicans* [45] and *T. crassiceps* [39], respectively. Regarding the role of complement in parasite damage, it is known that cysticerci are able to inhibit both classical and alternative pathways of the complement cascade, through C1q inhibition by parasite's paramyosins [46], but the relevance of the complement component 5 (C5) in the restrictiveness to parasite growth remains to be elucidated, especially considering that C5 is cleaved in two different active forms: C5a, which is a potent anaphylatoxin and chemotactic protein, and C5b, which has a binding site for C6 and is the molecule responsible for promoting the MAC (membrane attack complex) assembling in cell membrane. To assess the importance of C5 and possible involvement of additional genetic effects in the A/J versus B6 differential susceptibility to *T. crassiceps*, we classified the RCS according to susceptibility and C5 status (Table 1). In the majority of RCS, C5 status was a strong predictor of response to *T. crassiceps* infection with C5 deficiency causing increased parasite replication permissiveness, even when present on the B6 background as seen in BcA73, BcA70, BcA72 and BcA83 strains. Conversely, the presence of wild-type alleles at C5 on an otherwise permissive A/J background, as seen in AcB55 strain, conferred restrictiveness to infection. We noted the presence of several discordant strains (Table 1, denoted by an asterisk), such as BcA76, AcB60 and AcB63. Although C5 explains 25% of phenotypic variance, discordant strains suggest that additional susceptibility and resistance genetic factors, distinct from the dominant C5 effect, modulate response to *T. crassiceps* infection. In fact, STAT4 and STAT6 transcription factors were recently involved in modulation of the immune response to *T. crassiceps* in mice. Permissiveness of BALB/c mice was abrogated in STAT6^−/−^ mice of the same background, as they were able to efficiently mount a strong Th1 response and control the infection [47]. Conversely, Th1 response induced via STAT4-dependent signaling pathway is essential for development of immunity against cysticercosis [48]. Also, macrophage activation and subsequent production of nitric oxide represent additional mechanisms essential for host resistance to *T. crassiceps* infection [49]. Interestingly, we did not uncover any associations of the MHC with susceptibility in our AcB55xDBA/2 cross where DBA/2 mice carry parasite permissive H2-d [11]. This further suggests that the differential permissiveness observed in BALB/c susbstrains and attributed to H2-d haplotype is in fact, BALB-background specific. Similarly, the *Tccr2* locus represents a novel effect that arose upon combination of the AcB55 and DBA/2 genetic backgrounds and further emphasizes the importance of genetic modifiers in *T. crassiceps* permissiveness. Finally, generation of additional informative crosses will be necessary to validate and study the effect contributed by the chromosome 6 underlying genetic effect(s) in *T. crassiceps* cysticercosis, independently of C5 or in conjunction.

In conclusion, we have combined haplotype association mapping in 34 recombinant congenic strains and linkage analysis to identify and validate a novel locus that modulates the outcome to *T. crassiceps* infection in mice. We have demonstrated that C5 underlies the *Tccr1* locus and uncovered an important role of the complement pathway in susceptibility to *T. crassiceps* cysticercosis.

## Supporting Information

Figure S1 **Haplotype maps underlying EMMA-identified chromosome 2 and chromosome 6 associations in RCS.** Haplotype maps of permissive and restrictive RCS are depicted for the most significant associations on proximal chromosome 2 along with proximal and distal regions of chromosome 6, illustrating segregation of B6 allele (gray) with restrictiveness and A/J (white) allele with permissiveness to *T. crassiceps*.
(TIF)

Figure S2 **Parasite load in the [AcB55xA/J]F2 population.** [AcB55xA/J]F2 mice (n = 427) were infected intraperitoneally with 10 non-budding *T. crassiceps* larvae and parasite number was determined 30 days post-infection. The results from three separate infections are plotted along with A/J, B6, DBA/2, and [AcB55xA/J]F1 controls.
(TIF)

Figure S3 **Effect of the** ***Tccr2*** **locus on parasite burden in [AcB55xDBA/2]F2 mice.** Detailed LOD score traces are shown for chromosome 19 (*Tccr2*) locus for [AcB55xDBA/2]F2 females (**A**). The genome-wide thresholds are indicated at P = 0.01 and P = 0.05. The heterozygous-driven permissiveness to *T. crassiceps* in F2 mice is illustrated for *Tccr2* by segregating the parasite load according to the AcB55 (A), DBA/2 (D) or heterozygous (H) alleles at the rs13483650 peak SNP (**B**).
(TIF)
