# Supplementary figures and images for: Identification of Loci Controlling Restriction of Parasite Growth in Experimental Taenia crassiceps Cysticercosis
Source: PLoS Negl Trop Dis. 2011 Dec 20;5(12):e1435. doi: 10.1371/journal.pntd.0001435 (PMC3243719; doi:10.1371/journal.pntd.0001435)

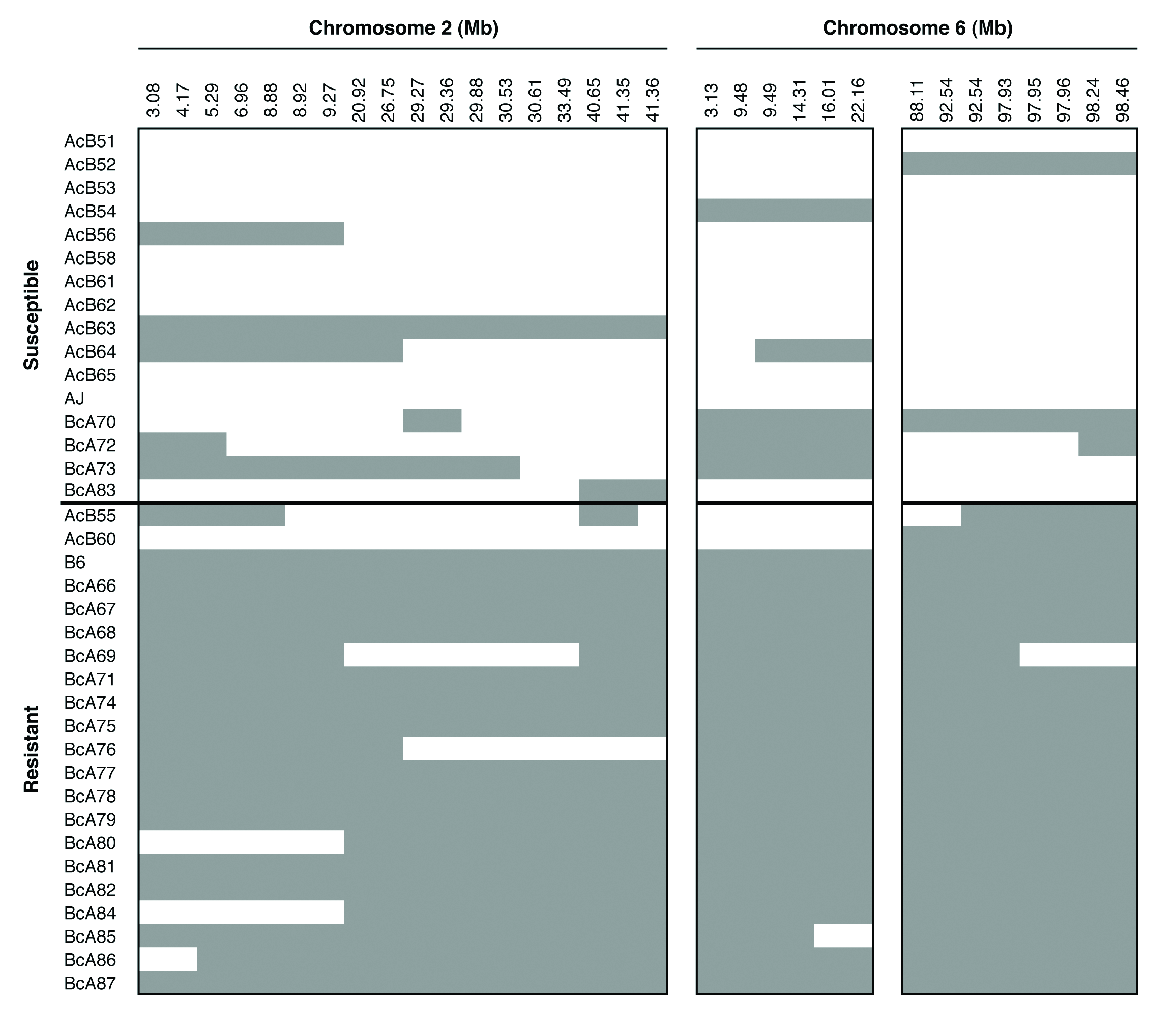

Supplement: Figure S1 — Haplotype maps underlying EMMA-identified chromosome 2 and chromosome 6 associations in RCS. Haplotype maps of permissive and restrictive RCS are depicted for the most significant associations on proximal chromosome 2 along with proximal and distal regions of chromosome 6, illustrating segregation of B6 allele (gray) with restrictiveness and A/J (white) allele with permissiveness to T. crassiceps. (TIF) [file pntd.0001435.s001.tif]

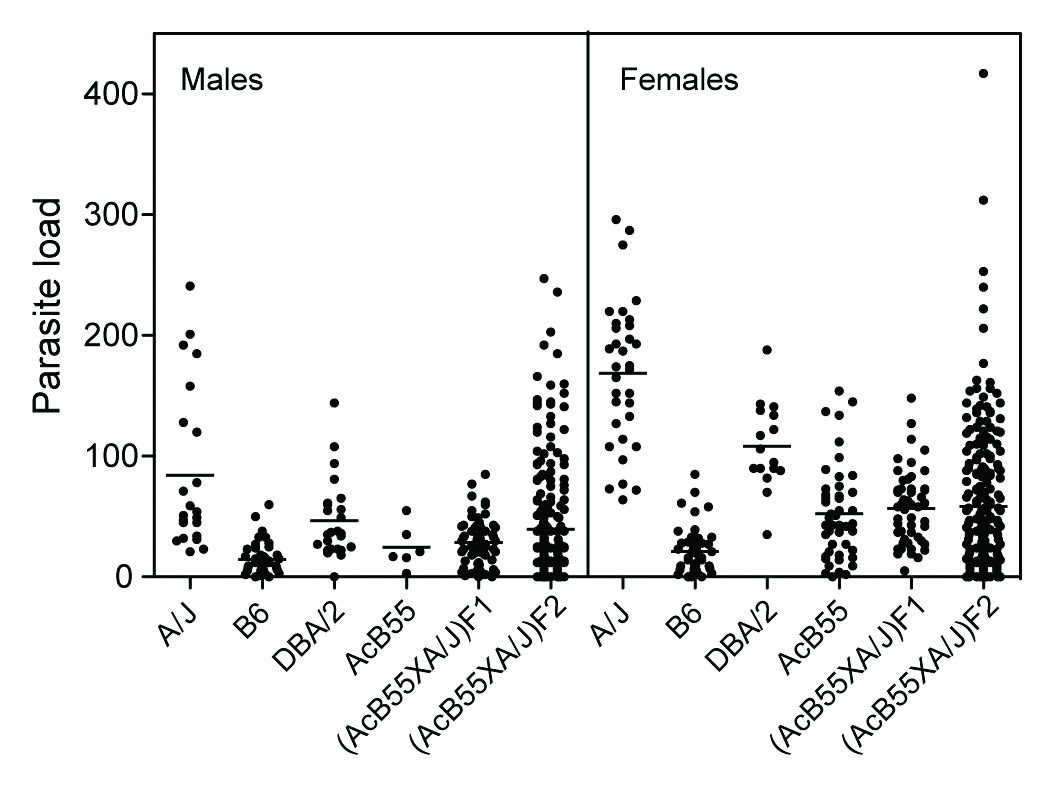

Supplement: Figure S2 — Parasite load in the [AcB55xA/J]F2 population. [AcB55xA/J]F2 mice (n = 427) were infected intraperitoneally with 10 non-budding T. crassiceps larvae and parasite number was determined 30 days post-infection. The results from three separate infections are plotted along with A/J, B6, DBA/2, and [AcB55xA/J]F1 controls. (TIF) [file pntd.0001435.s002.tif]

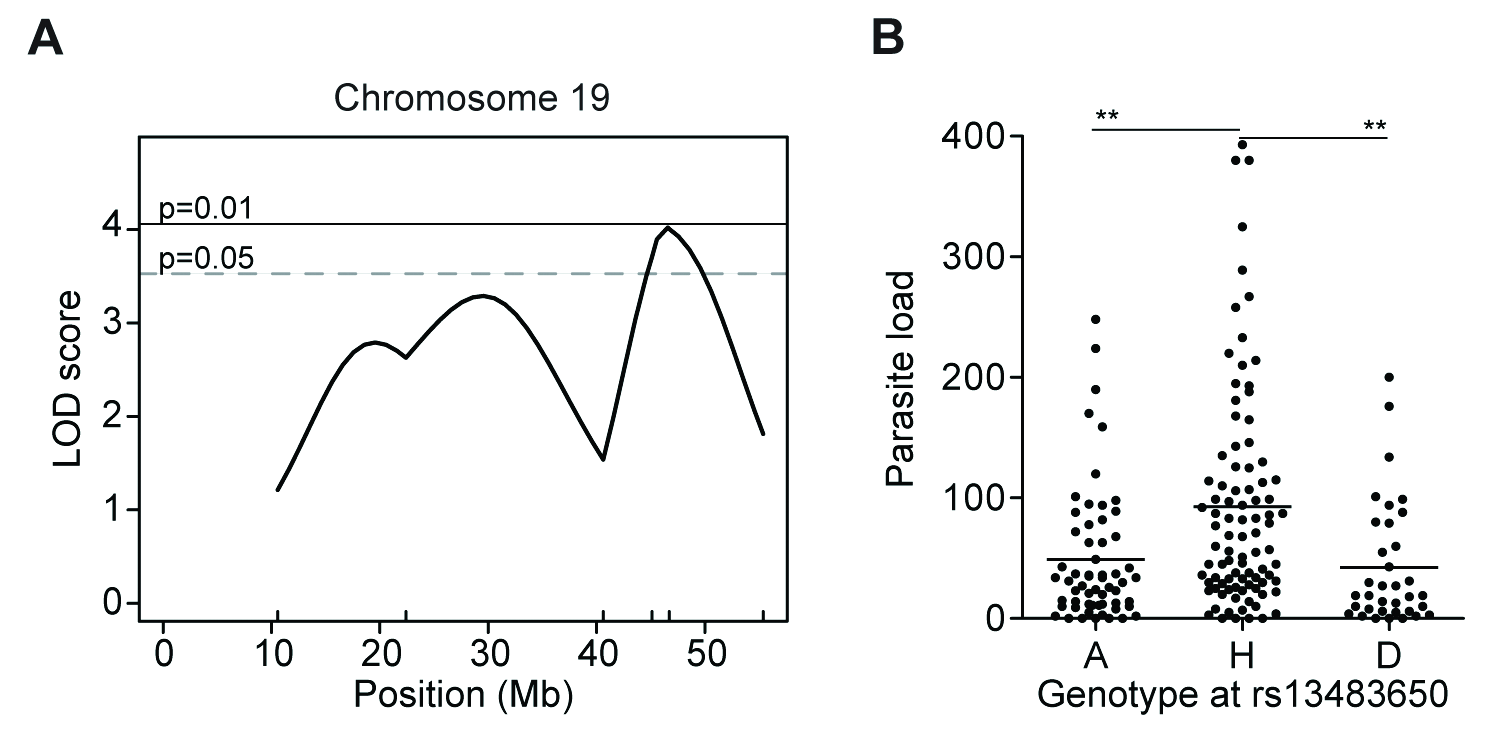

Supplement: Figure S3 — Effect of the Tccr2 locus on parasite burden in [AcB55xDBA/2]F2 mice. Detailed LOD score traces are shown for chromosome 19 (Tccr2) locus for [AcB55xDBA/2]F2 females (A). The genome-wide thresholds are indicated at P = 0.01 and P = 0.05. The heterozygous-driven permissiveness to T. crassiceps in F2 mice is illustrated for Tccr2 by segregating the parasite load according to the AcB55 (A), DBA/2 (D) or heterozygous (H) alleles at the rs13483650 peak SNP (B). (TIF) [file pntd.0001435.s003.tif]
